# New insights into the regulation of METTL3 and its role in tumors

**DOI:** 10.1186/s12964-023-01360-5

**Published:** 2023-11-23

**Authors:** Qiu Jin, Huinan Qu, Chengshi Quan

**Affiliations:** 1 The Key Laboratory of Pathobiology, Ministry of Education, College of Basic Medical Sciences, Jilin University, 126 Xinmin Avenue, Changchun, Jilin 130021 People’s Republic of China; 2 Department of Histology and Embryology, College of Basic Medical Sciences, Jilin University, 126 Xinmin Avenue, Changchun, Jilin 130021 People’s Republic of China

**Keywords:** METTL3, m^6^A, Cancer, Epigenetics

## Abstract

As one of the most abundant epigenetic modifications in RNA, N^6^-methyladenosine (m^6^A) affects RNA transcription, splicing, stability, and posttranscriptional translation. Methyltransferase-like 3 (METTL3), a key component of the m^6^A methyltransferase complex, dynamically regulates target genes expression through m^6^A modification. METTL3 has been found to play a critical role in tumorigenesis, tumor growth, metastasis, metabolic reprogramming, immune cell infiltration, and tumor drug resistance. As a result, the development of targeted drugs against METTL3 is becoming increasingly popular. This review systematically summarizes the factors that regulate METTL3 expression and explores the specific mechanisms by which METTL3 affects multiple tumor biological behaviors. We aim to provide fundamental support for tumor diagnosis and treatment, at the same time, to offer new ideas for the development of tumor-targeting drugs.

**Supplementary Information:**

The online version contains supplementary material available at 10.1186/s12964-023-01360-5.

## Background

m^6^A modification is the most abundant methylation modification of eukaryotic RNA [1–3] discovered in 1974 [4]. It can alter the stability of RNA, induce RNA conformational changes, regulate protein-RNA interactions, and manipulate microRNA maturation. Unlike the methylation modifications that occur at the 3’UTR or 5’Cap of RNA, the m^6^A modification deposited on the N6 position of RNA adenylate is highly selective and conserved as an internal RNA modification. Mostly, m^6^A modification occurs at the shared RNA motif of RRACH (R = A, G/U; R = A/G; H = A/U/C) [5]. m^6^A is mainly enriched in exons, near the stop codon, and at the 3’UTR. m^6^A modification is a dynamic and reversible process, mediated by the methyltransferase complex “writers”, the demethylase “erasers”, and the m^6^A binding protein “readers”.

Although m^6^A methyltransferase have been identified as a complex of several proteins, it was not until 1997 that MT-A70, a protein subunit of METTL3 with the methylation substrate S-adenosylmethionine (SAM), was first isolated from HeLa cells [6]. It is generally accepted that the m^6^A methyltransferase complex consists of seven evolutionarily conserved members, including METTL3, METTL14, WTAP, VIRMA, RBM15, ZC3H13 and Hakai.

METTL3, the core component of the catalytic methyltransferase complex, is recruited to the target RNA by the remaining components, using SAM as a donor to transfer methyl to the RRACH motif. METTL3 catalyzes the methylation of RNA, which further regulates the expression of target genes and influences cell biological behavior. Thus, METTL3 is involved in a wide range of physiological and pathological processes, such as haematopoiesis, immunity, viral infection and replication. In particular, METTL3 expression is elevated in a variety of tumor, enhancing the m^6^A levels of different target genes. The change in m^6^A content leads to altered expression of oncogenes/anti-oncogenes and activation of various pro-oncogenic signaling pathways. This study is dedicated to exploring the regulation of METTL3 and the specific mechanisms by which METTL3 affects tumor biological behavior, thus providing some reference for clinical development and application of drugs targeting METTL3.

## Structure and distribution of METTL3

The human METTL3 gene is located at 14q11.2 and contains 11 exons. The full-length METTL3 protein consists of 580 amino acids. The primary structure contains 20 different amino acids, with 58 leucine, 46 serine, 43 aspartic acid, 42 alanine, and 38 glutamic acid, forming a protein monomer with a molecular weight of 64 kDa.

**Fig. 1:**
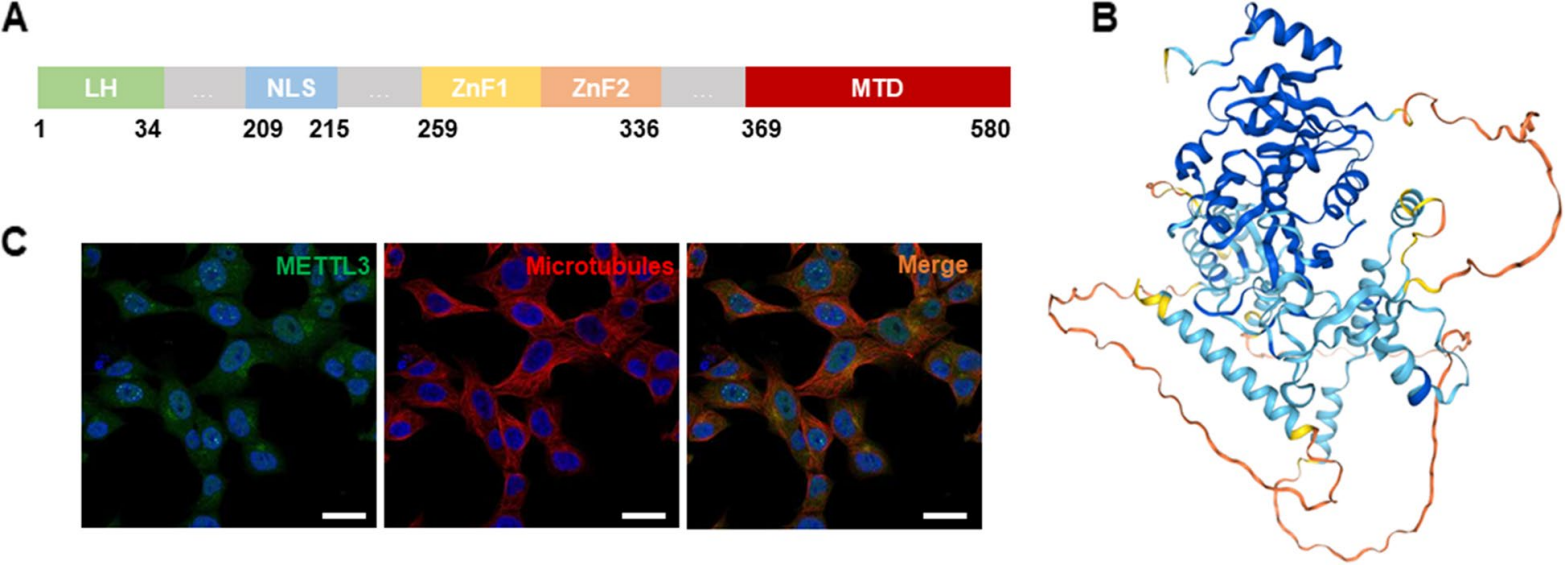
Structure and distribution of METTL3. **A** Schematic diagram of the two-dimensional structure of METTL3; **B** Structure prediction of METTL3 from the AlphaFold project; **C** HPA database showing the localization of METTL3 in HAP1 cells, bar = 20 μm

METTL3 is a highly conserved protein consisting of a leading helix (LH) (1–34), a nuclear localization signal (NLS) (209–215), a zinc finger domain (ZFD) and a methyltransferase domain (MTD) (369–580/357–580/358–580) (Fig. 1A, B). The LH and NLS of METTL3 work in concert with other members of the methyltransferase complex to facilitate the entry of METTL3 into the nucleus [7]. The ZFD in METTL3 consists of two CCCH-type zinc fingers, ZnF1 (259–298) and ZnF2 (299–336), which are responsible for the specific recognition of RNA and stabilization of the catalytic activity of methyltransferases [8]. The MTD of METTL3, the catalytic core of methylation, is a classical α-β-α sandwich fold consisting of four α helices (α1, α2, α4 on one side and α3 on the other) and eight β folds (in the order β1↑, β8↑, β7↑, β2↑, β3↑, β5↓, β4↑ and β6↑), three 310 helices and three loops with low sequence similarity, named gate loop 1 (residues 396–410), interface loop (residues 462–479) and gate loop 2 (residues 507–515).

Further studies showed that METTL3 contains one acetylation site Ser2 [9], eight phosphorylation sites Ser2, Ser43, Ser48, Ser50, Ser219, Ser243, Thr348, Ser350 [7] and four SUMOylation sites Lys177, Lys211, Lys212, and Lys21 [10].

In eukaryotes, the intracellular distribution of METTL3 varies among different cellular sources. METTL3 is predominantly found in the nucleus and a small amount of METTL3 is expressed in the cytoplasm [11] (Fig. 1C).

## How METTL3 mediates m^6^A modification

Sequence analysis shows that METTL3 belongs to the class I MTase family and has weak methyltransferase activity in vitro. Therefore, METTL3 needs to cooperate with other members of “writers” to exert high catalytic activity. The formation of the complex effectively enhances the METTL3-mediated enzymatic reaction leading to m^6^A modification of target RNAs.

Compared with METTL3 alone, the METTL3-METTL14 complex greatly enhances methyltransferase activity through synergistic effects [12]. METTL3 and METTL14 form a butterfly-like antiparallel heterodimer of approximately 40 Å width and 70 Å length in an asymmetric unit, interacting with extensive hydrogen bonding and producing a positively charged groove. The complex binds to SAM. Of these, SAM is only visible in the METTL3 pocket, demonstrating that METTL3 acts as the catalytic core, while METTL14 acts as the RNA-binding platform [13]. The residues 357–580 of METTL3 (MTD3) and the residues 111–456 of METTL14 (MTD4) interact with each other to form a stable complex. METTL14 stabilizes the METTL3 structure and interacts with the substrate RNA [14], facilitating the transfer of methyl groups from METTL3 to the target RNA.

Additionally, METTL3-METTL14 forms a complex with other cofactors to achieve full enzymatic activity. The METTL3-METTL14 heterodimer accumulates in the nuclear speckles and binds to the target RNA under the guidance of WTAP [7]. Next, the METTL3-METTL14-WTAP complex is recruited by VIRMA and mediates preferential RNA methylation near the 3’UTR and stop codons [15]. At the same time, METTL3 binds to RBM15 and recruits the methyltransferase complex to specific sites in the RNA. This results in selectively methylation of adjacent RRACH motifs in the target RNA while distant ones are ignored [16].

## Regulation of METTL3

The expression of METTL3 is regulated via several mechanisms, including gene activation, initiation of transcription, transcript modification and transport, transcript translation and posttranslational modifications. Among these, histone modification and DNA methylation modulate METTL3 gene activation. Transcription factors regulate the RNA level of METTL3 through transcription initiation. Noncoding RNAs then change the expression of METTL3 at the posttranscriptional level. Furthermore, posttranslational modifications such as phosphorylation alert the content of METTL3 at the protein level. In addition, some chemicals are involved in adjusting METTL3 expression, but the exact mechanism is not yet clear.

### Histone modifications

**Fig. 2:**
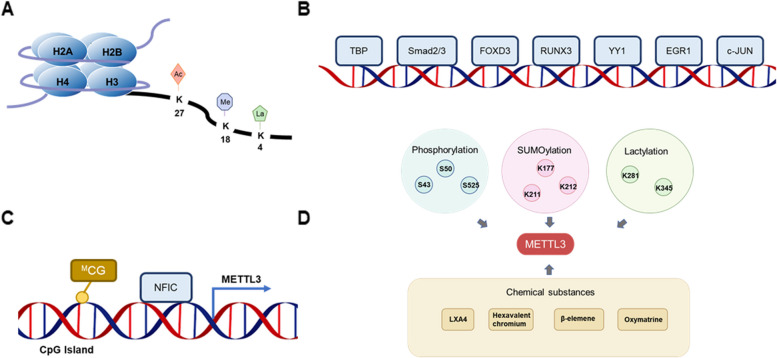
The regulation of METTL3. **A** Histone modifications occurring in the METTL3 promoter region. **B** Transcription factors regulating METTL3 transcription. **C** DNA methylation modifications regulate METTL3 expression. **D** Posttranslational modifications of METTL3 and some chemical substances regulating METTL3

In chromosomes, the proteins entangled in the DNA double strand are known as histones. Histones are octamers consisting of four components, H2A, H2B, H3 and H4. The N-terminal protein tails of the histone components have sites of modification such as methylation, acetylation and lactylation (Fig. 2A). Studies have shown that different modifications to the N-terminal protein tail of histones have different effects on the transcriptional activation of METTL3, which in turn affects the expression of METTL3.

In colorectal cancer, H3K4me3 is enriched in the METTL3 promoter region and promotes METTL3 expression through methylation [17]. In cervical cancer, WDR5 was found to mediate H3K4me3 histone modification of the METTL3 promoter and induce METTL3 transcriptional activation [18].

Wang et al. found abundant H3K27ac signals in the METTL3 promoter region in gastric cancer cells. Further experiments revealed that P300 mediated H3K27 acetylation of METTL3 and induced upregulation of METTL3 RNA expression at transcriptional level [19].

Xiong et al. found that METTL3 expression was elevated in tumor-infiltrating myeloid cells as lactate accumulated in the tumor microenvironment. Mechanistically, H3K18la, which is abundant in the METTL3 promoter region, elevates the expression of METTL3 by histone lactylation [20].

### DNA methylation

In multicellular eukaryotes, DNA methylation is the covalent bonding of a methyl group at the cytosine 5 carbon position of a genomic CpG dinucleotide in the presence of DNA methyltransferases [21]. Due to the CpG-rich island in the promoter region of the METTL3 gene, METTL3 can be transcriptionally regulated by DNA methylation.

Cigarette smoke condensate (CSC) decreases methylation within the CPG islets of the METTL3 gene. CSC increases transcription factor NFIC in the METTL3 promoter region and elevates METTL3 transcription in pancreatic ductal adenocarcinoma cells [22] (Fig. 2B).

### Transcription factors

Reportedly, multiple potential transcription factor binding sites exist in the METTL3 promoter region. In tumors, a variety of transcription factors have been identified that regulate the initiation of METTL3 transcription and its expression at the RNA level.

In cervical cancer cells, the transcription factor TBP binds directly to the promoter of METTL3 and upregulates METTL3 expression [23]. In gastric cancer, phosphorylated Smad2/3 is increased in the nucleus and initiates transcription of METTL3 [24]. In colorectal cancer, FOXD3 acts as a transcription activator to increase METTL3 expression [25]. Further studies revealed that RUNX3 interacts with the METTL3 promoter and activates circMETTL3 transcription in colorectal cancer [26]. In acute myeloid leukaemia, YY1 binds to the promoter of METTL3 by liquid-liquid phase separation, leading to elevated METTL3 expression [27]. In glioblastoma, EGR1 contributes to the high expression of METTL3 by binding straightly to the promoter of METTL3 [28]. In bladder cancer, activated c-JUN is recruited to the METTL3 promoter to enhance METTL3 transcription [29] (Fig. 2C).

### Noncoding RNA

Noncoding RNAs are a class of RNAs that do not encode proteins, including microRNAs (miRNAs), circular RNAs (circRNAs), tRNA-derived small RNA fragments (tRFs), and long noncoding RNAs (lncRNAs), etc. They can bind to the 3’UTR of target genes and affect gene expression. Noncoding RNAs are one of the important factors modulating METTL3 expression at the posttranscriptional level. For example, miR-302a directly targets the 3’UTR of METTL3 in M1-type macrophages and reduces the intracellular METTL3 RNA content [30]. In non-small cell lung cancer, circVMP1 acts as a sponge for miR-524-5p. circVMP1 releases METTL3 from the repression of miR-524-5p and enhances the protein expression of METTL3 [31]. In addition, the small RNA fragment tRF-1001 targets METTL3 and decreases the RNA level of METTL3 [32]. The effects of different noncoding RNAs on METTL3 are shown in the table below (Table 1).

**Table 1:** Effect of different noncoding RNAs on METTL3 expression

**Cell or Tissue**	**Source**	**Noncoding RNA**	**Effects**	**In vitro/vivo**	**Animal experiments**	**Reference**
Breast cancer	SUM-1315, MCF-7	miR-483-3p	Inhibition	In vitro	Not involved	[32]
	MCF-7	Let-7 g	Inhibition	In vitro	Not involved	[33]
	MDA-MB-231, BT-549	miR-34c-3p	Inhibition	In vitro	Not involved	[34]
Melanoma	SKMEL, HT-144	miR-302a-3p	Inhibition	In vitro	Not involved	[35]
Gastric cancer	AGS, MGC-803	miR-193b-5p	Inhibition	In vitro	Not involved	[36]
	HCG-27, MGC-803	miR-4429	Inhibition	In vitro	Not involved	[37]
	NCI-N87, SNU-16	miR-1269b	Inhibition	In vitro	Not involved	[38]
	SCG-7901, BGC-823	miR-338-5p	Inhibition	In vitro	Not involved	[39]
NSCLC	A549, NCI-H460	miR-33a	Inhibition	In vitro	Not involved	[40]
	A549, H1299	miR-600	Inhibition	In vitro	Not involved	[41]
	A549-S, A549-R	miR-4443	Inhibition	Both	Xenograft tumor model in nude mice	[42]
	A549, H1650	miR-590-5p	Inhibition	In vitro	Not involved	[43]
	H1299/DDP, A549/DDP	miR-524-5p	Inhibition	In vitro	Not involved	[30]
Hepatocellular carcinoma	HepG2, HuH-6	miR-186	Inhibition	Both	Xenograft tumor model in mice	[44]
	Hep3B	miR24-2	Promotion	In vitro	Not involved	[45]
	Hep3B	miR-1301-3p	Inhibition	In vitro	Not involved	[46]
Nasopharyngeal Carcinoma	KY-SE150, Eca-9706	miR-186-5p	Inhibition	In vitro	Not involved	[47]
M1 macrophages	LN‐229	miR-302a	Inhibition	In vitro	Not involved	[29]
Human Cartilage Tissues	Osteoarthritis Samples	miR-373	Inhibition	In vitro	Not involved	[48]
HRVEC		tRF-1001	Inhibition	Both	OIR mouse model	[49]
AML	Bone marrow samples of AML patients Molm13, HL-60	miR-493-5p	Inhibition	In vitro	Not involved	[32]

### Posttranslational modifications

The covalent binding of chemical groups or small molecules proteins to specific sites of amino acid sequences causes post-translational modification of proteins, which is essential for protein maturation and expression. Recent studies have shown that METTL3 is modified by phosphorylation, SUMOylation, and lactylation modifications.

ERK directly phosphorylates METTL3 at S43, S50 and S52. The zinc finger domain of ERK interacts with USP5 to reduce the level of ubiquitination-mediated degradation of METTL3, enhancing the stability of METTL3 [50]. Activated ataxia-telangiectasia mutated (ATM) kinase also upregulates METTL3 expression by phosphorylation [51].

SUMOylated sites K177, K211, K212 and K215 have been identified on the amino acid sequence of METTL3. The SUMOylation of METTL3 does not affect METTL3 expression, localization or binding to other methyltransferase complex components, but inhibits METTL3 methyltransferase activity [10]. Furthermore, Xv et al. found that the SUMO-conjugating enzyme E2 UBC9 promoted SUMO1-mediated SUMOylation of METTL3. Decreased methyltransferase activity of SUMOylated METTL3 results in reduced intracellular m^6^A content [52] (Fig. 2D).

In addition, the K281 and K345 sites of METTL3 can be directly modified by lactylation, which allows METTL3 to acquire stronger RNA binding capacity and promote m^6^A methylation of target RNAs [20].

### Chemical substances

A variety of chemical substances, such as fatty acids and metal contaminants, are involved in regulating METTL3 expression. However, the underlying mechanism by which chemicals alter METTL3 expression is unclear. Endogenous arachidonic acid LXA4, a small lipid molecule secreted by prostate cancer cells, significantly inhibits the RNA and protein expression of METTL3 in mouse peritoneal macrophages [53]. Chronic hexavalent chromium exposure upregulates METTL3 expression in mouse and human lung tumors [54]. In addition, in lung cancer, β-elemene targets and inhibits METTL3 expression at both RNA and protein levels [55]. In oral squamous cell carcinoma, Oxymatrine targets METTL3 and suppresses its expression [56] (Fig. 2D).

## Role of METTL3 in tumor cell proliferation

**Fig. 3:**
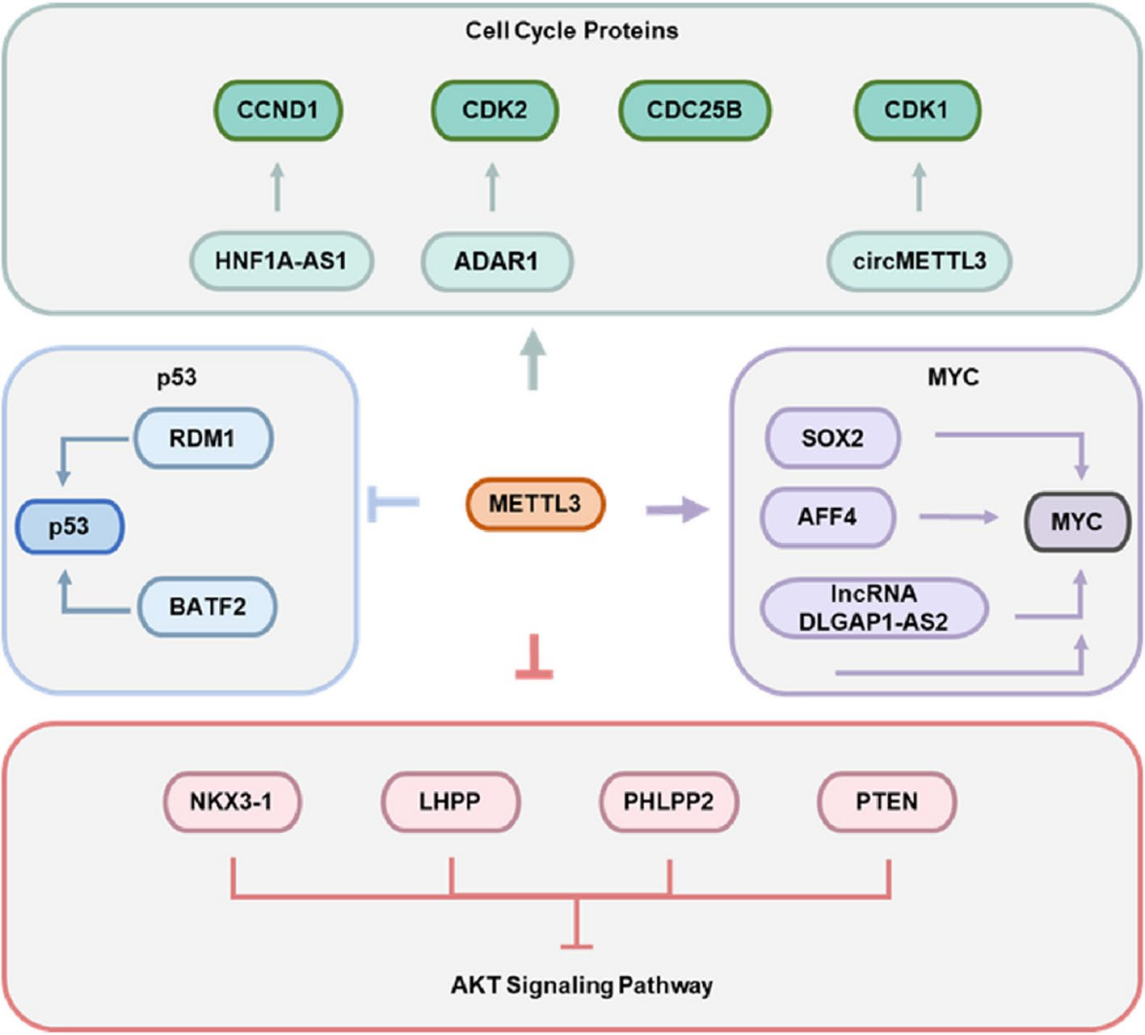
Specific mechanisms by which METTL3 regulates tumor cell proliferation

One of the fundamental features of tumors is the unlimited proliferative potential of tumor cells. The continuous release of growth signals within tumor cells promotes the cell cycle process and induces mitosis. Activation of oncogenes and inactivation of tumor suppressor genes accelerate cell cycle progression by regulating the distribution of growth signals. In a wide range of tumors, METTL3 relies on m^6^A modification to regulate the expression of classical oncogenes such as AKT and MYC to promote cell proliferation. Moreover, classical tumor suppressor genes such as p53 are under-expressed in tumors due to the negative regulation of m^6^A modification mediated by METTL3. Furthermore, METTL3 directly regulates the expression of cell cycle proteins and affects tumor growth (Fig. 3).

### AKT signaling

AKT is a serine/threonine kinase that is a central node of many signaling pathways and can phosphorylate a variety of downstream proteins. A wide range of growth signals can activate AKT, and then activated AKT promotes proliferation and inhibits apoptosis. AKT has been previously reported to be modified by various posttranslational modifications, such as O-GlcNAcylation, SUMOylation, acetylation, and ubiquitination [57, 58]. Currently, METTL3-mediated m^6^A regulation of AKT signaling is of interest.

In endometrial cancer, reduced expression of METTL3 leads to increased proliferation and tumorigenicity. Mechanistically, reduced m^6^A mediated by METTL3 results in diminished expression of the AKT negative regulator PHLPP2 and increased expression of the AKT positive regulator mTORC2. These results identify METTL3 as a regulator of AKT signaling [59].

However, the regulation of AKT activity by METTL3 varies in different tumors. In bladder cancer, knockdown of METTL3 significantly inhibits proliferation in vivo and in vitro. This may be associated with the significant upregulation of the tumor suppressors LHPP and NKX3-1 at both the RNA and protein levels, which inhibits the phosphorylation of downstream AKT [60]. In uveal melanoma, METTL3 induces AKT phosphorylation and promotes cell cycle progression by upregulating the expression of the target gene c-Met via m^6^A [61]. In pancreatic cancer, METTL3 synergistically induces SMS expression with IGF2BP3 and promotes AKT phosphorylation, thus enhancing tumor cell proliferation [62].

In bladder cancer, METTL3 accelerates pri-miR221/222 maturation through m^6^A modification, which downregulates PTEN expression through miR221/222 binding to its 3’UTR and stimulates proliferation in vitro and in vivo [63].

METTL3 in ovarian cancer cells suppresses PTEN expression by accelerating miR-126-5p maturation. The decrease of PTEN content leads to the activation of PI3K/AKT signaling pathway, which in turn elevates phosphorylated AKT and its downstream effectors driving ovarian cancer growth [64]. The same mechanism is also found in lung cancer [42], retinoblastoma [65], and esophageal cancer [66].

In summary, the regulation of AKT signaling by METTL3-mediated m^6^A plays an important role in tumor cell proliferation, but the mechanisms involved remain to be further explored.

### MYC regulation

MYC is a powerful oncogene that drives tumorigenesis and encodes a member of the bHLH-zip transcription factors that act as master transcription factors. In human cancers, dysregulated expression of MYC greatly promotes tumor cell proliferation.

Through m^6^A, METTL3 can either directly upregulate MYC expression or enhance the expression of AFF4, the transcription promoter of MYC, to regulate the expression of MYC in bladder cancer [67]. In non-small cell lung cancer, METTL3 enhances lncRNA DLGAP1-AS2 stability via m^6^A modification and interacts with YTHDF1 to improve MYC RNA stability [68]. In colorectal cancer, METTL3 cooperates with IGF2BP2 to increase the stability of SOX2 RNA, which promotes the transcription of MYC and enhances the self-renewal and proliferation of tumor cells [69].

Interestingly, the mechanism by which METTL3 enhances MYC expression in an m^6^A-dependent manner to regulate tumor growth also exists in cervical cancer [70], bladder cancer [71], acute myeloid leukaemia [72], and oral squamous carcinoma [73].

As a transcription factor, MYC is mainly located in the nucleus and lacks a specific small molecule active site. Therefore, it is difficult to inhibit its activity or to target MYC with specific monoclonal antibodies. Thus, studies on the regulatory effect of METTL3 on MYC expression, mediated either directly or indirectly by m^6^A, in the context of tumor growth are expected to provide a new therapeutic strategy for tumors.

### p53 influence

As a classical tumor suppressor, wild-type p53 monitors the integrity of genes and regulates cell cycle progression. Once cellular DNA is damaged, the p53 protein binds to the corresponding part of the gene and inhibits the activity of the cell cycle proteins, arresting the cell in G1 phase and thus inhibiting malignant proliferation. It is now believed that p53 is regulated by METTL3 in different ways, which affects tumor growth.

METTL3, which is highly expressed in hepatocellular carcinoma cells, inhibits RDM1 expression by increasing the m^6^A modification of its RNA. In fact, RDM1 binds to p53, which enhances p53 protein stability and inhibits phosphorylation activation of the Ras/Raf/ERK pathway to induce G2/M cell cycle arrest and impair the capability of cell proliferation [74]. Similarly, METTL3 reduces the expression of the p53-binding protein BATF2 via m^6^A modification to accelerate the growth of gastric cancer. Specifically, the reduced binding of BATF2 to p53 induced downregulates the stability of p53 and inhibits the phosphorylation activation of ERK in gastric cancer cells [75].

### Cell cycle protein impact

In addition to manipulating p53 expression levels, METTL3 also affects mitosis in eukaryotic cells by regulating the expression of cell cycle proteins.

The expression of METTL3 is significantly increased in the M-phase of cervical cancer cells. Enhanced m^6^A modification mediated by METTL3 accelerates translation of CDC25B and increases the proportion of tumor cells in G2/M stage, leading to malignant cancer growth [76]. The same mechanism is also observed in head and neck squamous cell carcinoma [77]. In glioblastoma, ADAR1 elevates CDK2 expression by binding to CDK2, while METTL3 upregulates ADAR1 protein expression and promotes G1/S phase transition [78]. In breast cancer, METTL3, a host gene for circMETTL3, regulates circMETTL3 expression in an m^6^A-dependent manner. CircMETTL3 is a competitive endogenous RNA of miR-31-5p that upregulates the expression of its target gene CDK1 and promotes tumor cell proliferation [79].

In colorectal cancer, METTL3 regulates long chain noncoding RNA HNF1A-AS1 expression, accelerating cell cycle progression and promoting proliferation. Mechanistically, HNF1A-AS1 enhances CCND1 expression by inhibiting PDCD4 or competitively sponging miR-93-5p [80].

METTL3 directly modulates the cell cycle to promote tumor cell proliferation, which undoubtedly provides a new perspective in our understanding of malignant tumor proliferation.

## Role of METTL3 in tumor cell migration and invasion

**Fig. 4:**
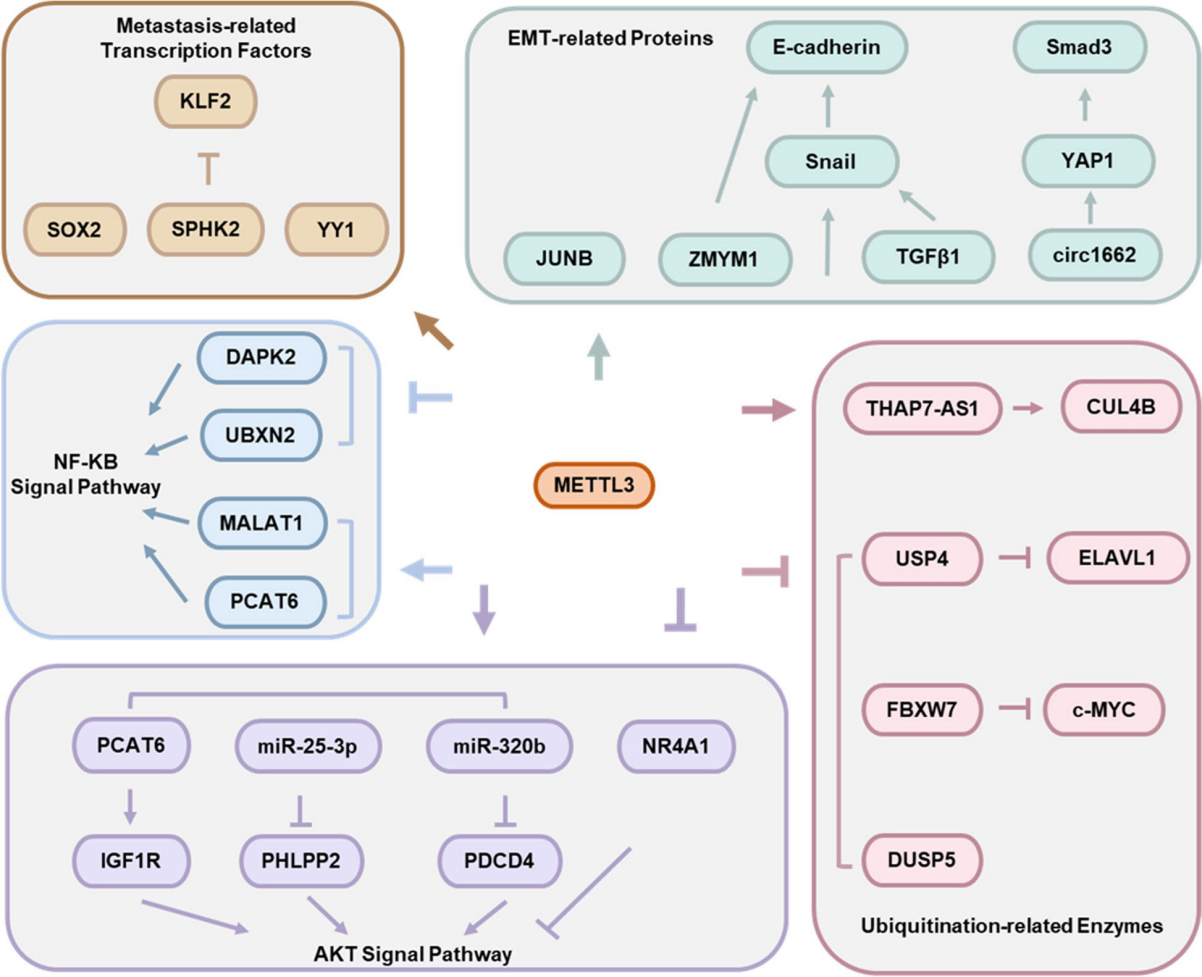
Specific mechanisms by which METTL3 regulates tumor metastasis

Due to genetic instability and heterogeneity, metastatic tumors physically disseminate from the primary site to distant tissues via capillaries or capillary lymphatics. Furthermore, metastatic tumors tolerate microenvironments that are not conducive to their growth and accomplish a variety of biological behaviors in the new microenvironment [81]. Significantly high expression of METTL3 was observed in a variety of metastatic tumors [82]. Mechanistically, METTL3 regulates the expression of epithelial-mesenchymal transition (EMT)-related genes through m^6^A modification. Moreover, METTL3 activates metastasis-associated oncogenic signaling pathways and upregulates related transcription factors. In addition, METTL3 regulates the ubiquitination of metastasis-related target genes, thereby promoting tumor metastasis (Fig. 4).

### EMT- related proteins

EMT is an important step in cancer cell metastasis and is essentially characterized by the loss of E-cadherin. Snail, Slug, Twist, Zeb1, and Smad3 regulate E-cadherin expression as EMT-related transcription factors. TGFβ acts as a prominent inducer of EMT development.

Lin et al. found that METTL3 inhibited E-cadherin expression by regulating Snail splicing to promote Snail translation and enhance protein stability, which affected EMT in nasopharyngeal carcinoma [83]. Furthermore, Xv et al. demonstrated that SUMOylated METTL3 enhanced m^6^A modification to improve Snail RNA stability and expression, which promoted EMT progression in hepatocellular carcinoma cells [52]. In gastric cancer cells, METTL3 increases Snail and Slug expression to repress E-cadherin transcription, promoting the occurrence of EMT and tumor metastasis [24]. Interestingly, Li et al. found that METTL3 could induce EMT by promoting TGFβ1 dimer formation, activating the TGFβ1/Smad2/Snail signaling pathway [84].

JUNB is an important transcriptional regulator of EMT. In lung cancer, METTL3 affects the RNA expression and stability of JUNB through m^6^A modification, which stimulates the EMT process [29].

In colorectal cancer, expression of the non-coding RNA circ1662 is upregulated by METTL3-mediated m^6^A modification. Highly expressed circ1662 straightly binds to YAP1 and contributes to the accumulation of YAP1 in the nucleus. YAP1 negatively regulates Smad3 expression to mediate EMT and tumor metastasis [85]. Pan et al. further showed that METTL3 induced CRB3 degradation through m^6^A modification. Reduced CRB3 altered the state of the Hippo pathway, promoting YAP nuclear localization and EMT in colorectal carcinoma [86].

In gastric cancer, METTL3 cooperates with HuR to enhance the RNA and protein stability of ZMYM1 in an m^6^A-dependent manner. ZMYM1 recruits the CtBP/LSD1/CoREST complex to the E-cadherin promoter and suppresses E-cadherin expression through physical association, modulating EMT and metastasis [87]. These results suggest that METTL3 plays a vital role in EMT in tumor cells.

### Classical oncogenic signaling pathways

In addition, the activation of some classical oncogenic signaling pathways such as NF-KB and AKT is essential for cancer metastasis.

In gliomas, METTL3 induces the m^6^A modification and degradation of UBXN2 RNA in concert with YTHDF2, activating the downstream NF-KB signaling pathway and boosting tumor metastasis [88]. Additionally, METTL3-mediated m^6^A modification enhances the stability and expression of the oncogenic lncRNA MALAT1. MALAT1 promotes NF-KB phosphorylation and nuclear ectopic expression, which in turn activates the NF-KB signaling pathway [89]. In non-small cell lung cancer, METTL3 downregulates DAPK2 expression, leading to enhanced tumor migration in vitro and in vivo following the activation of the NF-KB signaling pathway [90]. In bladder cancer, METTL3 upregulates PCAT6 expression and increases IGF1R RNA stability by forming the PCAT6/IGF2BP2/IGF1R complex [91], which activates the NF-KB and PI3K/AKT signaling pathways.

In pancreatic ductal adenocarcinoma, METTL3 accelerates the maturation of the miR-25-3p precursor. miR-25-3p targets and represses the expression of PHLPP2, which contributes to tumor metastasis by activating the downstream AKT-p70S6K signaling pathway [22]. Similarly, in esophageal squamous cell carcinoma, METTL3 enhances the maturation of miR-320b via m^6^A modification to downregulate the expression of PDCD4. The miR-320b-PDCD4 axis activates the AKT signaling pathway to drive tumor metastasis [92]. In cervical cancer, METTL3 induces m^6^A modified NR4A1 RNA degradation through the YTHDF2-DDX6 pathway to manipulate tumor metastasis. In detail, the role of NR4A1 in recruiting the LSD1/HDAC1/CoREST complex to the AKT1 promoter is weakened, and the transcriptional activity of AKT1 is promoted [93]. In papillary thyroid cancer, METTL3 stabilizes STEAP2 RNA and positively regulates STEAP2 expression in an m^6^A-dependent manner, which inhibits the Hedgehog signaling pathway and suppresses cancer metastasis [94].

Interestingly, in medulloblastoma, METTL3 acts directly on PTCH1 and GLI2, important factors in the Hedgehog pathway, to promote tumor progression [95]. The above results suggest that METTL3 also affects tumor metastasis by regulating important metastasis-related signaling pathways.

### Metastasis-related transcription factors

Moreover, METTL3 relies on m^6^A modification to regulate some important metastasis-related transcription factors and promote the metastasis of various cancers.

In concert with IGF2BP2, METTL3 promotes m^6^A modification of the SOX2 CDS region to inhibit SOX2 RNA degradation, which increases the protein content of intracellular SOX2 and induces colorectal cancer metastasis [69].

Furthermore, METTL3 enhances SPHK2 expression to increase KLF2 ubiquitination-mediated degradation and promote malignant gastric cancer progression [96].

In multiple myeloma, METTL3 increases YY1 expression and promotes tumor progression by enhancing the RNA stability of YY1 [97].

It is thus suggested that the regulatory role of METTL3 in tumor metastasis is not limited to classical metastasis-related proteins and signaling pathways.

### Ubiquitination-related enzymes

In addition, the manipulation of ubiquitination-mediated degradation of multiple tumor metastasis-related genes by METTL3 to regulate tumor cell migration and invasion has attracted attention.

In bladder cancer, knockdown of METTL3 significantly inhibited cell migration and invasion. Mechanistically, METTL3 mediates the m^6^A modification of the deubiquitinating enzyme USP4 RNA at A2696, which in turn promotes the binding of YTHDF2 and HNRNPD to USP4 RNA, leading to USP4 degradation. The reduction in USP4 expression decreases the level of ELAVL1 protein deubiquitination, leading to decreased ELAVL1 protein expression and increased ARHGDIA expression, promoting bladder cancer cell migration and invasion [98].

In lung adenocarcinoma, significantly low expression of METTL3 inhibits m^6^A modification and translation of the E3 ubiquitin ligase FBXW7, resulting in reduced levels of ubiquitination and degradation of oncogenes such as MYC, accelerating tumor metastasis [99].

In gastric cancer, METTL3 enhances the expression of THAP7-AS1 by m^6^A modification to improve the migration and invasion capacity of tumor cells. Mechanistically, THAP7-AS1 facilitates E3 ubiquitin ligase CUL4B protein entry into the nucleus to suppress miR-22-3p and miR-320a expression and activate the PI3K/AKT signaling pathway [100].

The above studies suggest that METTL3 relies on m^6^A to regulate the expression of multiple tumor metastasis-associated proteins and activate oncogenic signaling pathways. However, whether crosstalk exists between these signaling pathways and the participants remains to be investigated in depth.

## Role of METTL3 in tumor aerobic glycolysis

**Fig. 5:**
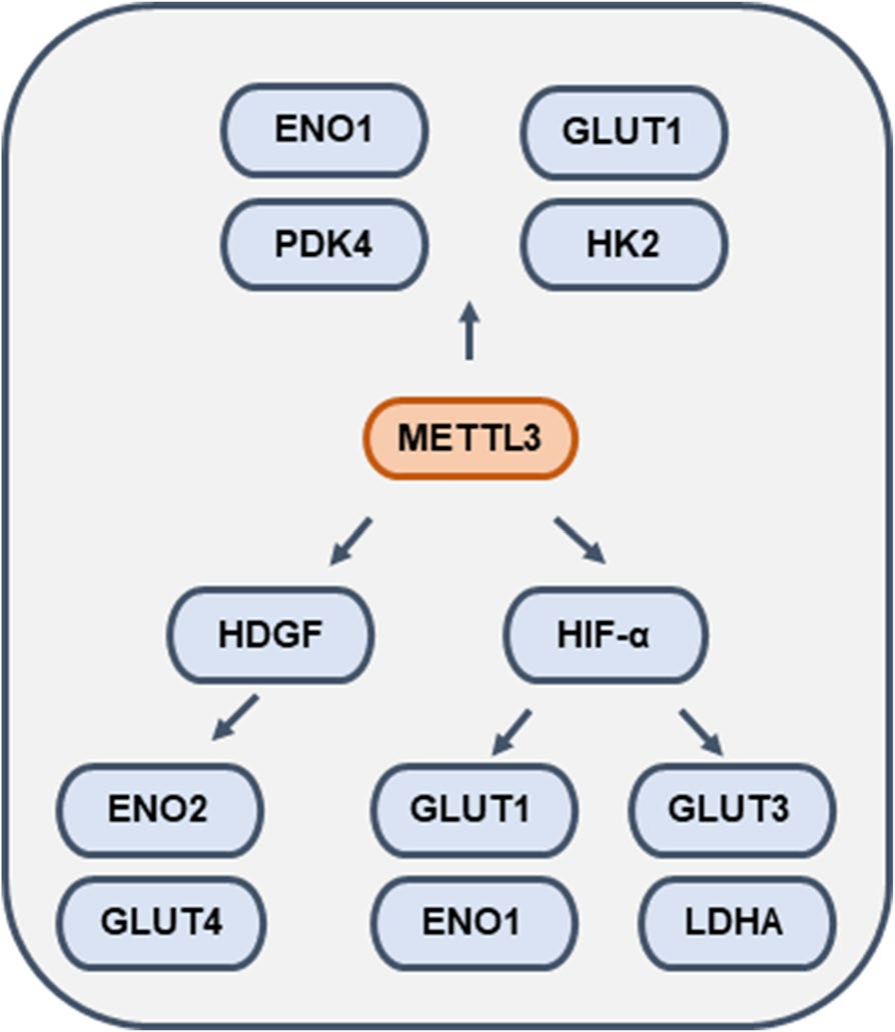
Mechanism of METTL3 regulation of metabolic reprogramming in tumors

Aerobic glycolysis is one of the key hallmarks of tumors. On the one hand, aerobic glycolysis provides nutrients and energy to replenish the enormous energy gap needed for tumor growth and metastasis, permitting tumor cells to gain a competitive advantage in a threatened microenvironment; On the other hand, metabolites themselves, such as lactate, can be carcinogenic by altering cell signaling and preventing cell differentiation [101]. It is now believed that METTL3 regulates the metabolic reprogramming of tumors by modulating the expression of glucose transporters (GLUTs), lactate dehydrogenase (LDHA) and enolase 1 (ENO1) in tumor cells (Fig. 5).

In gastric cancer, high METTL3 expression significantly elevates glucose uptake and lactate production. Mechanistically, METTL3 enhances the stability of HDGF RNA in an m^6^A-dependent manner to promote HDGF entry into the nucleus. Nuclear HDGF acts as a transcription factor and induces the expression of target genes GLUT4 and ENO2, which enhances glycolysis and promotes tumor growth [19]. In hepatocellular carcinoma, METTL3 facilitates GLUT1 and GLUT3-mediated glycolysis through the upregulation of HIF1α [102]. Consistent with this, in gastric cancer, METTL3 enhances the stability of NDUFA4. NDUFA4 upregulates the expression of the HIF1-α target genes ENO1 and LDHA to promote glucose uptake, then increases ECAR and OCR as well as the cellular lactate and ATP levels [103]. METTL3 increases PDK4 RNA stability and translation to promote ATP production and glycolysis in cervical cancer cells [23]. In addition, METTL3 also acts directly on key glycolytic enzymes to regulate metabolic reprogramming. In colorectal cancer, METTL3 stabilizes HK2 and GLUT1 to activate aerobic glycolysis pathway via the deposited m^6^A in the 3’UTR/5’UTR [104]. In lung adenocarcinoma, METTL3-mediated m^6^A modification of ENO1 at 359A stimulates glycolysis and tumorigenesis [105].

Exploration of the role of METTL3 in tumor glycolysis facilitates the elucidation of the specifics of energy metabolism within tumor cells, which undoubtedly broadens our view of metabolic reprogramming in tumors.

## Role of METTL3 in tumor immune escape

**Fig. 6:**
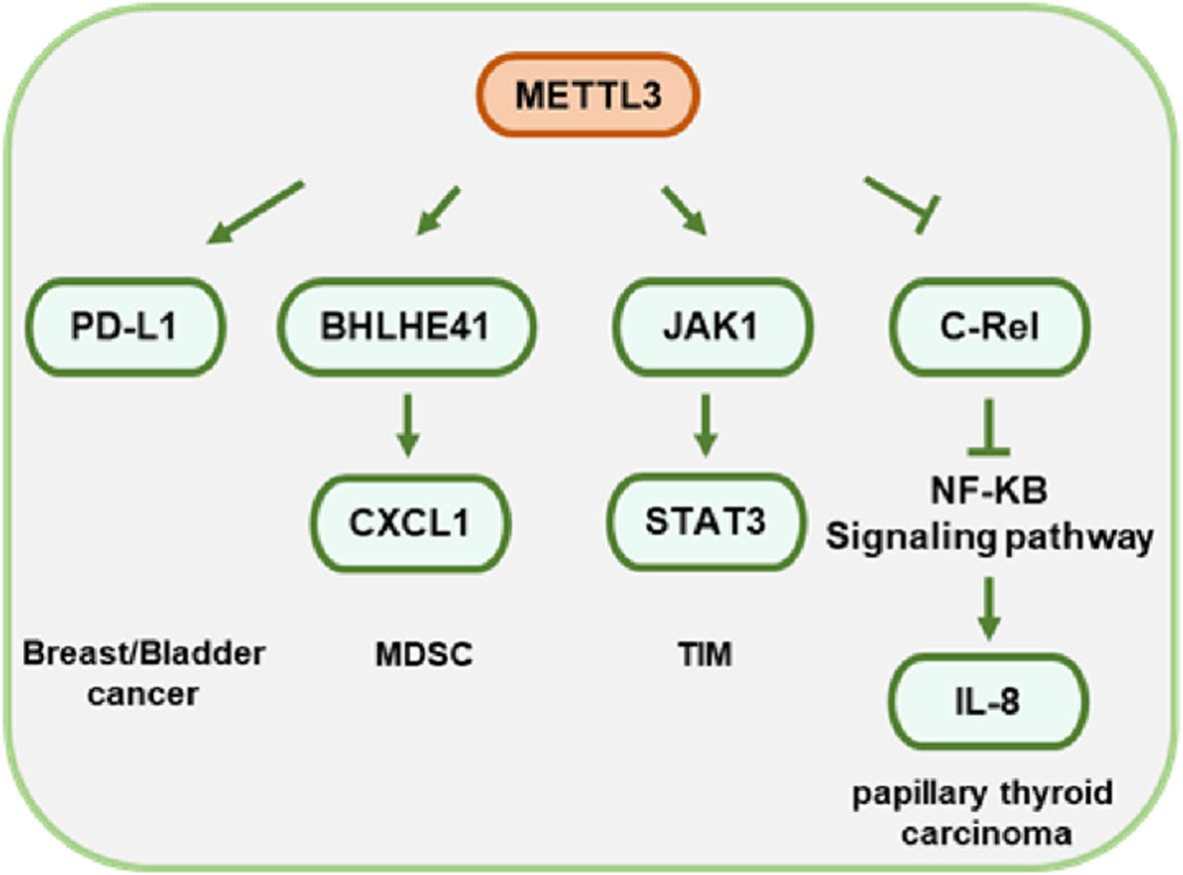
Role of METTL3 in tumor immune escape

Tumor microenvironment is an indispensable part of tumor immune escape. The metabolic profile of tumor cells induces a hypoxic, hypoglycaemic and acidic tumor microenvironment that shifts the function of immune cells and cytokines from a tumor suppressive to a tumor promoting state. These alteration leads to tumor immune escape rather than the establishment of an effective host anti-tumor response. Most tumors shape the tumor microenvironment to promote tumor immune escape by recruiting immunosuppressive cells such as myeloid-derived suppressor cells (MDSCs), regulatory T cells (Tregs) and tumor-associated macrophages (TAMs), or by activating immunosuppressive signaling pathways such as those involving PD1/PDL1.It has been found that METTL3 plays an important role in tumor immune escape (Fig. 6).

In colorectal cancer, METTL3 depletion inhibits colorectal tumorigenesis. Silencing METTL3 exerts an inhibitory effect on the accumulation of MDSCs to maintain the activation and proliferation of CD4+ and CD8+ T cells. Mechanistically, METTL3 promotes the expression of BHLHE41 in an m^6^A-dependent manner and subsequently induces CXCL1 transcription to enhance MDSC migration in vitro [106]. Pathological tissue analysis of cervical cancer likewise showed that METTL3 expression positively correlates with the level of CD33+ MDSCs [107].

Meanwhile, in tumor-infiltrating myeloid cells (TIMs), METTL3 mediates the m^6^A modification of JAK1 RNA and enhances the translation efficiency of the JAK1 protein, followed by the activation of STAT3 phosphorylation to promote tumor cell growth [20].

In bladder cancer, m^6^A is enriched in the 3’UTR of PD-L1 RNA. High expression of METTL3 enhances PDL1 RNA stability and expression level, which facilitates resistance to CD8+ T-cell toxicity and boosts tumor immune escape in vitro and in vivo [108]. Similarly, METTL3 enhances immune escape by upregulating PDL1 expression and inhibiting the activation of antitumor T cells in breast cancer [109].

However, in papillary thyroid carcinoma, METTL3 directly destabilizes c-Rel mRNA by increasing m^6^A levels and, together with YTHDF2, inactivates the NF-KB pathway and increases IL-8 secretion to induce neutrophil infiltration [110]. The effect of METTL3 on the immune microfluidic response of the immune system has been discussed. The role of METTL3 in the immune microenvironment and immune infiltration remains to be further investigated, but there is no doubt that targeting METTL3 therapy is a novel means to overcome the challenge of tumor immune escape.

## The role of METTL3 in tumor drug resistance

**Fig. 7:**
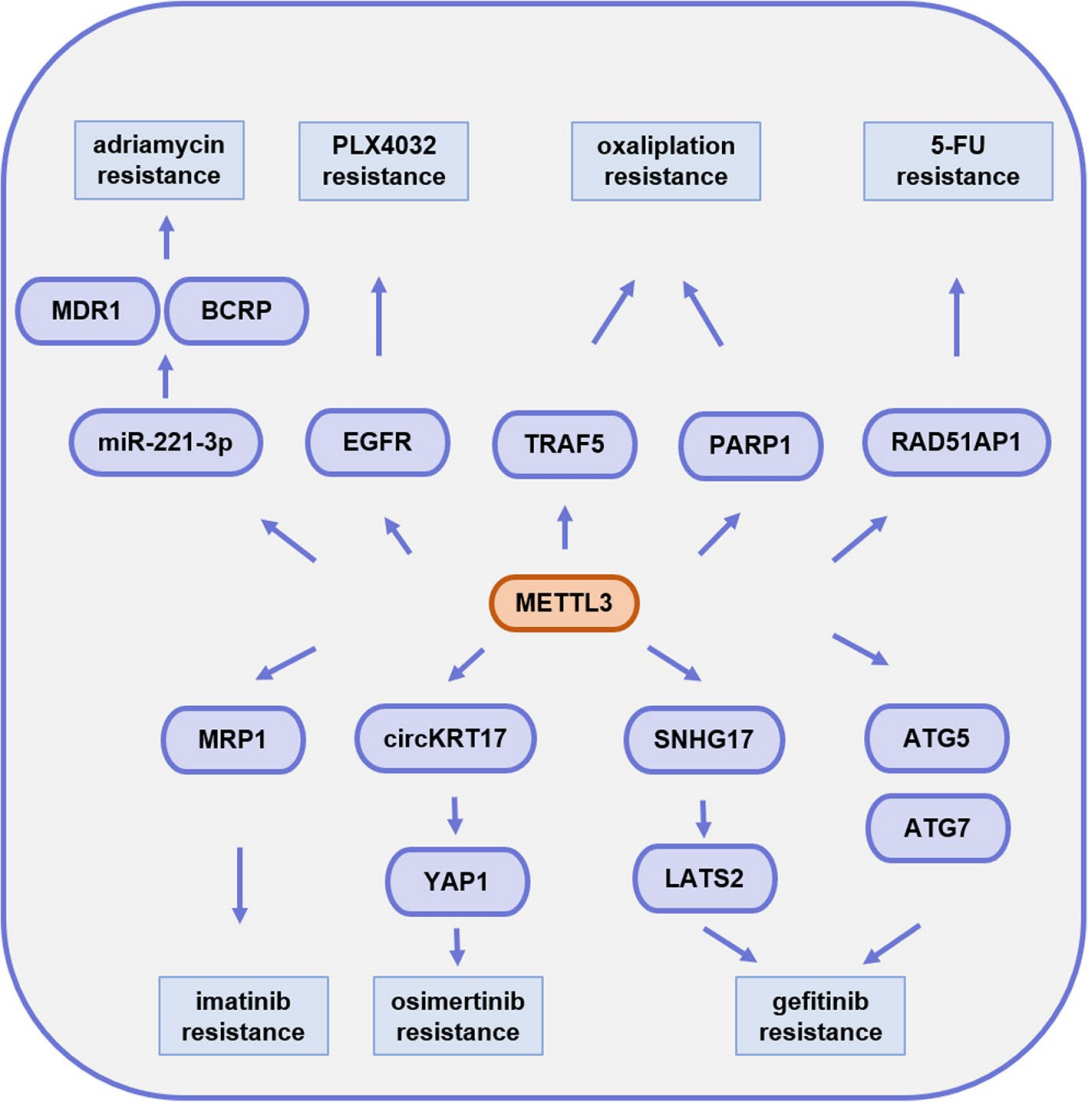
METTL3 regulates tumor cell drug resistance through different pathways

The causes of drug resistance in tumor cells are complex, with the involvement of genetic mutations, altered pharmacokinetics, activation of classical signaling pathways, and altered cellular adaptations. METTL3 relies on m^6^A modification to affect tumor cell drug resistance at multiple levels, including expression of anticancer drug targets and multidrug transporter proteins, classical signaling pathway switches, cellular antioxidant effects, DNA damage repair, cellular autophagy, and apoptosis (Fig. 7).

EGFR is an important cancer driver critical for tumor growth and survival. EGFR is a commonly used drug target in clinical practice. In melanoma, METTL3 elevates the m^6^A modification level of EGFR mRNA and increases its translational efficiency. High expression of EGFR activates the RAF/MEK/ERK pathway and induces resistance to PLX4032 in tumor cells [111].

In imatinib-resistant gastrointestinal stromal tumors, ETV1 activates METTL3 transcription and further promotes m^6^A modification mediated by METTL3 at the 5’UTR of MRP1 RNA, stimulating translation of MRP1 RNA and promoting imatinib resistance in tumor cells [112]. In breast cancer, METTL3 accelerates miR-221-3p precursor maturation, induces MDR1 and BCRP expression via the miR-221-3p/HIPK2/Che-1 axis, then promotes adriamycin resistance in cancer cells [113].

METTL3 and circKRT17 levels are elevated in osimertinib-insensitive lung adenocarcinoma tissues and cells. METTL3 enhances circKRT17 expression by promoting m^6^A modification. When it is overexpressed, circKRT17 recruites EIF4A3 to enhance YAP1 stabilization and nuclear import, upregulating osimertinib resistance in tumor cells [114]. In addition, METTL3 increases the RNA stability and expression of SNHG17 via m^6^A modification in gefitinib-resistant lung adenocarcinoma. Specifically, SNHG17 recruits EZH2 to the promoter of LATS2, epistemically repressing LATS2 and inducing gefitinib resistance [115].

One of the principles of chemotherapeutic drugs for tumor treatment is that they induce the production of reactive oxygen radicals in tumor cells, which rapidly depletes the antioxidant system, causing DNA damage and inducing programmed and nonprogrammed tumor death. In colorectal cancer, METTL3 inhibits TRAF5 expression by reducing TRAF5 stability. Downregulated intracellular TRAF5-mediated necrosis leads to enhanced antioxidant effects and increased resistance to oxaliplatin in tumor cells [116]. Moreover, METTL3 promotes resistance to 5-FU by upregulating the expression of RAD51AP1. Specifically, increased binding of RAD51AP1 to RAD51 results in an elevated ability to repair damaged DNA strands [117]. METTL3 enhances PARP1 RNA stability, which heightens the activity of the base excision repair pathway and effective repair of oxaliplatin-induced DNA damage in tumor cells, further promoting oxaliplatin resistance in CD133+ gastric cancer stem cells [118].

METTL3 induces the expression of genes such as ATG5 and ATG7 in gefitinib-resistant non-small cell lung cancer and regulates autophagy to promote drug resistance in tumor cells [119]. In addition, METTL3 induces autophagy to enhance the imatinib resistance in gastrointestinal stromal tumor cells by upregulating USP13 expression and promoting deubiquitination of ATG5 [120].

Tumor drug resistance has always been a risk factor for the prognosis of tumor patients. In-depth studies on METTL3 affecting the expression of various drug resistance-related genes through m^6^A modification can help us better understand the mechanism of tumor drug resistance and provide new therapeutic strategies for chemotherapy-resistant tumor patients.

## Drug development and clinical treatment based on METTL3

**Fig. 8:**
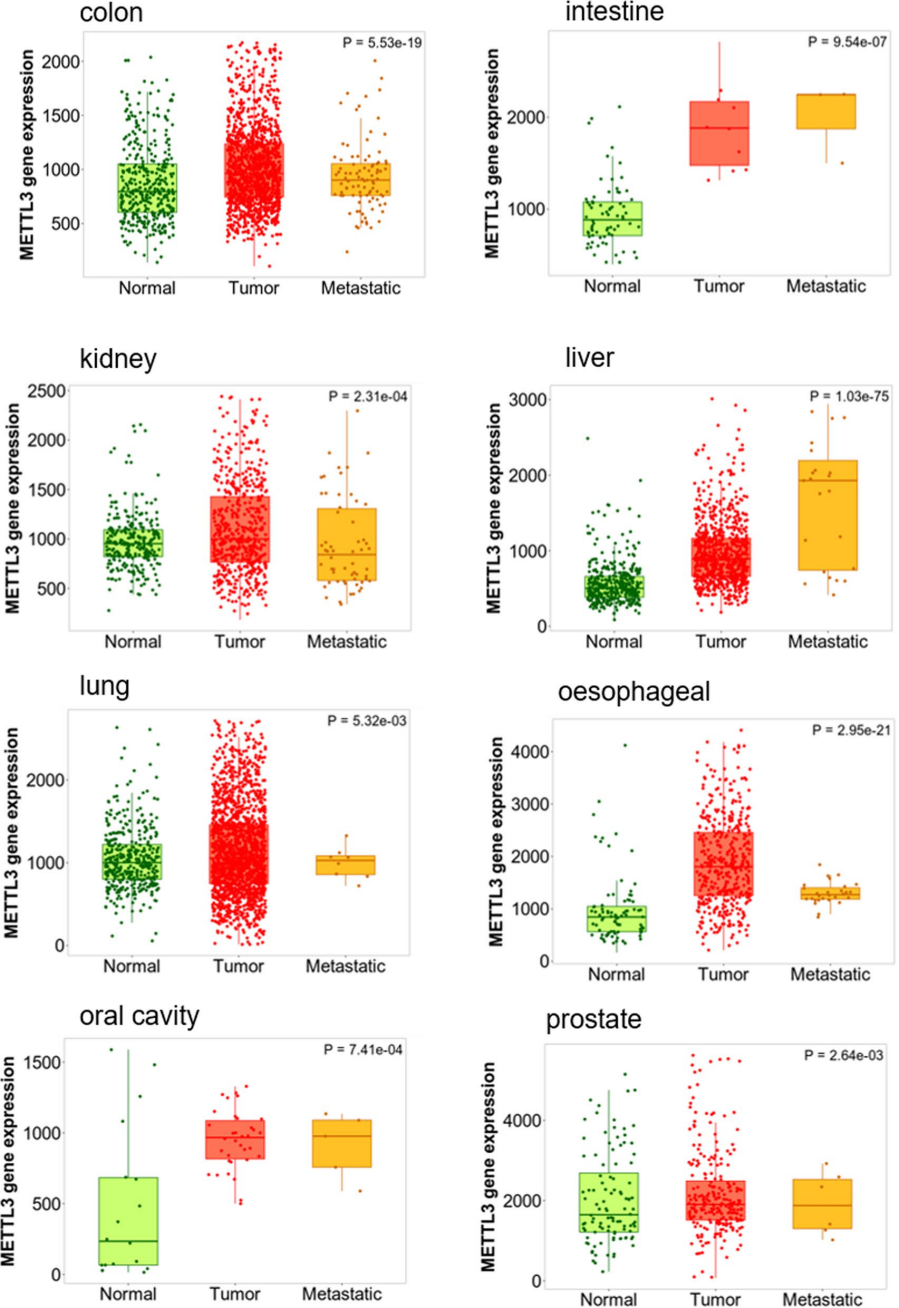
Expression of METTL3 in different tissue types (normal, tumor and metastatic) from GeneChip data analyzed using TNMplot database

METTL3, which is highly expressed in various primary and metastatic tumor tissues (Fig. 8), plays a crucial role in tumors through m^6^A modification. Consequently, several inhibitors targeting METTL3 have emerged, which undoubtedly provides new insights for tumor therapy. METTL3 requires a methyl group as a donor for m^6^A modification, therefore, effective reduction of METTL3 activity through competitive binding of small molecule complexes has become one of the ideas for the development of METTL3 inhibitors.

Substrate competitive inhibitors of METTL3 can be divided into nucleoside analogues and nonnucleoside analogues. However, despite the ability of nucleoside analogues to inhibit METTL3, they have low cell permeability and poor analytical selectivity, so research has progressively focused on the development of non-nucleoside analogues.

The currently disclosed nonnucleoside analogues, including UZH1a, UZH2 and STM2457, all exhibit high selectivity for METTL3 and have high inhibition efficiency. Among them, STM2457 has demonstrated excellent antitumor activity and therapeutic efficacy in vivo in AML patient-derived tumor models. Treatment of AML cell lines with STM2457 significantly inhibited tumor growth and increased the differentiation and apoptosis of key stem cell subpopulations. In vivo treatment of METTL3 with STM2457 is a promising therapeutic strategy for AML [121].

Moreover, the allosteric inhibitor 43n shows high selectivity and potent enzyme inhibitory activity for the METTL3-METTL14 complex based on the reversibility of the allosteric sites and the noncompetitive inhibition function [122].

Given the wide distribution of METTL3 in normal and tumor tissues, therapeutic agents targeting METTL3 may be of great clinical value and general applicability.

## Conclusions

The expression and biological function of METTL3, one of the core catalases for m^6^A modification, have been studied extensively in recent years. Unsurprisingly, METTL3 is a crucial hub in tumor growth and progression, regulating the splicing, stability, and expression of a wide range of genes through m^6^A modifications. Trace it to its cause, the regulation of genes by METTL3 is multilayered. METTL3 can not only directly alter effector genes expression, but also indirectly manipulate effector genes by affecting upstream regulators or signaling pathways.

Flexible regulation enables METTL3 to function efficiently in tumors. As a result, it is extremely urgent and important to explore the factors that regulate the expression and role of METTL3 in tumor cells. Here, we summarize several factors affecting METTL3, such as histone modifications, DNA methylation, noncoding RNAs, transcription factors, and several posttranslational modifications. These results provide new ideas for us to gain insights into the mechanism by which METTL3 promotes tumor progression and, at the same time, provide certain clues for the development of anticancer drugs.

Drug development targeting METTL3 is undoubtedly promising, as METTL3 is highly expressed in a wide range of tumors. According to the structure of METTL3 and its mechanism of action, the following strategies have been proposed in drug development studies: (1) development of SAM competitive inhibitors based on the principle of substrate competitiveness; (2) inhibition of the methyltransferase activity of the MTD structural domain of METTL3; (3) disruption of the binding of METTL3 to METTL14; and (4) manipulation of the entry of METTL3 into the nucleus. Recently, several drugs developed based on the principle of substrate competitiveness have demonstrated certain effects. In the future, the pool of drugs targeting METTL3 will be greatly enriched.

However, it is worth noting that METTL3 is widely present in tissues, and drugs targeting METTL3 should have high tissue specificity; otherwise, they may have an impact on the biological behavior of normal tissues.

In summary, although the specific roles and mechanisms of METTL3 in different types of tumors require more detailed and comprehensive research, the treatment of tumors targeting METTL3 is an undoubtedly promising strategy. With further research, METTL3 may become a molecular marker for tumor diagnosis as well as a treatment target.

## Data Availability

Not applicable.

## Abbreviations

m^6^A: N6-methyladenosine
METTL3: Methyltransferase-like 3
SAM: S-adenosylmethionine
METTL14: Methyltransferase 14
WTAP: WT1 associated protein
VIRMA: Vir like m6A methyltransferase associated
RBM15: RNA binding motif protein 15
ZC3H13: Zinc finger CCCH-type containing 13
LH: Leading helix
NLS: Nuclear localization signal
ZFD: Zinc finger domain
MTD: Methyltransferase domain
CSC: Cigarette smoke condensate
TBP: TATA-box binding protein
Smad2/3: SMAD family member 2/3
FOXD3: Forkhead box D3
RUNX3: RUNX family transcription factor 3
YY1: YY1 transcription factor
EGR1: Early growth response 1
miRNAs: MicroRNAs
circRNAs: Circular RNAs
tRFs: TRNA-derived small RNA fragments
lncRNAs: Long noncoding RNAs
PHLPP2: PH domain and leucine rich repeat protein phosphatase 2
LHPP: Phospholysine phosphohistidine inorganic pyrophosphate phosphatase
NKX3-1: NK3 homeobox 1
IGF2BP3: Insulin-like growth factor 2 mRNA binding protein 3
CDC25B: Cell division cycle 25B
ADAR1: Adenosine deaminase RNA specific 1
PDCD4: Programmed cell death 4
RDM1: RAD52 motif containing 1
BATF2: Basic leucine zipper ATF-like transcription factor 2
AFF4: ALF transcription elongation factor 4
SPHK2: Sphingosine kinase 2
ZMYM1: Zinc finger MYM-type containing 1
JUNB: AP-1 transcription factor subunit
DAPK2: Death-associated protein kinase 2
UBXN2: UBX domain-containing protein 2
MALAT1: Metastasis-associated lung adenocarcinoma transcript 1
PCAT6: Prostate cancer associated transcript 6
NR4A1: Nuclear receptor subfamily 4 group A member 1
ELAVL1: ELAV-like RNA binding protein 1
USP4: Ubiquitin specific peptidase 4
FBXW7: F-box and WD repeat domain containing 7
IGF1R: Insulin-like growth factor 1 receptor
ENO1: Enolase 1
GLUT1: Solute carrier family 2 member 1
HK2: Hexokinase 2
PDK4: Pyruvate dehydrogenase kinase 4
LDHA: Lactate dehydrogenase A
HDGF: Heparin binding growth factor
HIF1-α: Hypoxia inducible factor 1 subunit alpha
PDL1: Programmed cell death 1 ligand 1
BHLHE41: Basic helix-loop-helix family member e41
CXCL1: C-X-C motif chemokine ligand 1
C-Rel: Reticuloendotheliosis oncogene
IL-8: Interleukin 8
EGFR: Epidermal growth factor receptor
TRAF5: TNF receptor associated factor 5
PARP1: Poly (ADP-ribose) polymerase 1
RAD51AP1: RAD51-associated protein 1
MRP1: ATP binding cassette subfamily C member 1
SNHG17: Small nucleolar RNA host gene 17
LATS2: Large tumor suppressor kinase 2
ATG5: Autophagy related 5
ATG7: Autophagy related 7
